# Detection and molecular characterization of urinary tract HIV-1 populations

**DOI:** 10.1186/s12941-019-0326-9

**Published:** 2019-09-24

**Authors:** M. L. Mzingwane, G. Hunt, R. Lassauniere, M. Kalimashe, A. Bongwe, J. Ledwaba, R. E. Chaisson, N. Martinson, K. Richter, S. M. Bowyer, C. T. Tiemessen

**Affiliations:** 1 Department of Pathology, Faculty of Medicine, National University of Science & Technology, Ascot, P. O Box AC 939, Bulawayo, Zimbabwe; 2 Department of Medical Virology, University of Pretoria, Pretoria, South Africa; 3 Centre for HIV and Sexually Transmitted Infections, National Institute for Communicable Diseases, Johannesburg, South Africa; 4 Faculty of Health Sciences, University of the Witwatersrand, Johannesburg, South Africa; 5 Virus Research and Development Laboratory, Department of Virus and Microbiological Special Diagnostics, Statens Serum Institut, Copenhagen, Denmark; 6 Johns Hopkins University Center for AIDS Research, Baltimore, MD USA; 7 Perinatal HIV Research Unit (PHRU), SA MRC Soweto Matlosana Collaborating Centre for HIV/AIDS and TB, University of the Witwatersrand, Johannesburg, South Africa; 8 National Health Laboratory Services Tshwane Academic Division, Pretoria, South Africa

## Abstract

**Background:**

Identification of all possible HIV reservoirs is an important aspect in HIV eradication efforts. The urinary tract has however not been well studied as a potential HIV reservoir. In this pilot study we molecularly characterized HIV-1 viruses in urine and plasma samples to investigate HIV-1 replication, compartmentalization and persistence in the urinary tract.

**Methods:**

Prospectively collected urine and blood samples collected over 12–36 months from 20 HIV-1 infected individuals were analysed including sampling points from prior to and after ART initiation. HIV-1 *pol* gene RNA and DNA from urine supernatant and urine pellets respectively were analysed and compared to plasma RNA viruses from the same individual.

**Results:**

HIV-1 nucleic acid was detected in urine samples from at least one time point in 8/20 (40%) treatment-naïve subjects compared to 1/13 (7.7%) individuals on antiretroviral treatment (ART) during periods of plasma viral suppression and 1/7 (14.3%) individuals with virological failure. HIV-1 RNA was undetectable in urine samples after ART initiation but HIV-1 DNA was detectable in one patient more than 6 months after treatment initiation. There was co-clustering of urine-derived *pol* sequences but some urine-derived sequences were interspersed among the plasma-derived sequences.

**Conclusions:**

Suppressive ART reduces HIV-1 replication in the urinary tract but HIV-1 DNA may persist in these cells despite treatment. A larger number of sequences would be required to confirm HIV compartmentalization in the urinary tract.

## Introduction

Persistent HIV reservoirs harbouring integrated, replication-competent proviral DNA serve as a source of viral rebound on interruption of antiretroviral therapy (ART), resulting in a requirement for lifelong ART. Independent replication and selection pressures at different anatomic sites coupled with restricted genetic exchange may result in distinct compartmentalized variants [1]. Determining the anatomic reservoirs responsible for persistent viremia and viral rebound and the types of cells involved is important in the current efforts for HIV eradication and identification of targets for new drugs.

Lymph nodes have been shown to harbour replication-competent virus in virally suppressed individuals [2]. Other tissues and organs including the gut, male and female genital tracts and central nervous system have also been identified as potential sources of HIV-1 persistence [3–6]. We have previously reviewed the mechanisms of HIV persistence during ART in these reservoirs [7]. However some potential reservoirs such as the urinary tract, which includes the urethra, bladder and kidneys, have not been well studied. There is a wide spectrum of HIV-associated renal disease including HIV-associated nephropathy and HIV immune-complex kidney disease, the pathogenesis of which has been linked to HIV-1 infection of renal epithelium [8, 9]. In African countries the prevalence of renal disease associated with HIV ranges from 6 to 45% [10]. HIV infection of renal tubular epithelial cells in patients on suppressive antiretroviral therapy has been reported [11–13], and HIV infection of kidney allografts after transplantation has also been detected in HIV positive recipients with undetectable HIV virus in plasma at the time of transplantation [14]. These findings highlight the kidneys as a potential HIV reservoir in virally suppressed individuals on ART. In this pilot study, we isolated and molecularly characterized HIV-1 viruses in urine samples collected prospectively from twenty HIV-1 infected individuals, before and after antiretroviral treatment initiation and compared them to blood-derived viruses to investigate HIV-1 replication, compartmentalization and persistence in the urinary tract.

## Methods

### Patients and samples

Twenty HIV-1 positive individuals were selected from the parent study, the Soweto Lung Cohort (n = 756) [15] recruited from patients in Soweto, South Africa between November 2008 and October 2012. This sub-study restricted participants to those with detectable viremia after at least 6 months on antiretroviral treatment initiation. Participants had scheduled 6 monthly clinic visits where blood and urine samples and clinical information were collected (Additional file 1: Table S1). Viral loads and CD4 counts were tested at each visit. If local eligibility criteria for ART initiation were met, patients were referred for initiation of ART. Virological failure was defined as VL > 1000 RNA copies/ml 6 months after starting ART. A total of 97 urine samples from 20 patients were identified and analysed from the archived specimens. Following collection, urine samples were clarified by centrifugation and stored at − 70 °C as supernatants and cell pellets. Virions were concentrated from 2 ml stored urine supernatant by high-speed centrifugation (25,000 rpm for 2 h) and the supernatant removed leaving ~ 200 µl for HIV-1 RNA extraction. Urine pellets were re-suspended in 200 µl phosphate buffered saline before HIV-1 proviral DNA extraction.

### HIV-1 amplification and *pol* gene sequencing

Total nucleic acid was extracted from 200 µl of sample and eluted in 50 µl of elution buffer using the automated NucliSENS easyMAG extraction system (Biomerieux, Boxtel, The Netherlands) with external lysis, and stored at − 20 °C until required for nucleic acid amplification. Extracted DNA was quantitated using the NanoDrop 2000 Spectrophotometer (Thermo Scientific). Genotyping of a 1084 base pair (bp) region of the HIV-1 *pol* gene covering the protease (*PR*) gene and codon 1–250 of the reverse transcriptase (*RT*) gene was performed using a one step reverse transcription polymerase chain reaction (RT-PCR) method [16]. Sequences were edited and assembled on Sequencher V 4.5 (Gene Codes Corporation, USA) and submitted to the Stanford HIVdb algorithm V 7.0. (http://hivdb.stanford.edu/) for subtyping, interpretation of resistance mutations and quality assessment. RNA and DNA viruses from urine samples were compared to RNA viruses from plasma samples detected from the same individual using phylogenetic analysis. Alignments were performed in MEGA6 and manually edited in BioEdit v 7.2.5 and neighbour joining trees constructed under the Kimura 2-parameter model with 1000 bootstrap values.

## Results

### Characteristics of subjects

Samples of 20 subjects meeting eligibility criteria were tested for the presence of HIV-1 nucleic acid in their longitudinal urine samples (Table 1). Study participants were followed up for between 12 and 36 months with a mean follow up period of 27 months (std: 8.81 months). Median CD4 count and plasma viral load at enrolment was 265 cells/µl and 4.17 log_10_ RNA copies/ml, respectively. Eighteen (90%) individuals had progressive HIV-1 disease defined as CD4 decline to < 300 cells/µl of whom 16 were initiated on ART during the study period. The other two individuals initiated ART at CD4 counts of 526 and 420 cells/µl.

**Table 1:** Clinical and demographical characteristics of 20 study participants

	Study participants
Age (years)	
Mean (std)	37.8 (6.4)
Gender	
% males	3 (15%)
% females	17 (85%)
Log_10_ viral load at enrolment	
Median (IQR)	4.17 (4.05–4.44)
CD4 count at enrolment (cells/µl)	
Median (IQR)	265 (232–325)
CD4 count declined to < 300 during follow up	18 (90%)
Initiated ART during study	
N	18 (90%)
Treatment failure	
N	7 (35%)
N participants followed up for (months)	
12	1 (5%)
18	7 (35%)
24	1 (5%)
30	3 (15%)
36	8 (40%)

### Prevalence of HIV-1 nucleic acid detection in urine samples

HIV nucleic acid was detected in 27/97 (13.9%) of all urine samples representing 8 (40%) individuals (Table 2). Both HIV-1 RNA and proviral DNA were detected in urine supernatant and urine pellets, respectively, from 7 of the subjects, while only HIV-1 RNA was detected in the urine supernatant from subject 051.

**Table 2:** HIV-1 nucleic acid detection in urine samples

Group	Positive samples			# of subjects with positive samples		
	Supernatant	Pellet	Total	Supernatant	Pellet	Any
Overall Samples n = 97 Subjects n = 20	13 (13.4%)	14 (14.4%)	27 (13.9%)	8 (40%)	7 (35%)	8 (40%)
Treatment naïve Samples n = 84 Subjects n = 20	12 (14.3%)	13 (15.5%)	25 (15.2%)	8 (40%)	7 (35%)	8 (40%)
Virological failure (VL > 1000 RNA copies/ml) Samples n = 13 Subjects n = 7^a^	0 (0%)	1 (7.7%)	1 (3.8%)	0 (0%)	1 (14.3%)	1 (14.3%)
Virally suppressed/low level viremia (VL < 1000 RNA copies/ml) Samples n = 21 Subjects n = 13^b^	1 (4.8%)	0 (0%)	1 (2.4%)	1 (7.7%)	0 (0%)	1 (7.7%)

^a^Subject IDs with VL > 1000 RNA copies/ml: 038, 051, 100, 121, 662, 664, 732

^b^Subject IDs with VL < 1000 RNA copies/ml: 030, 051, 074, 100, 121, 614, 236, 747, 658, 664, 668, 705, 732

**Fig. 1:**
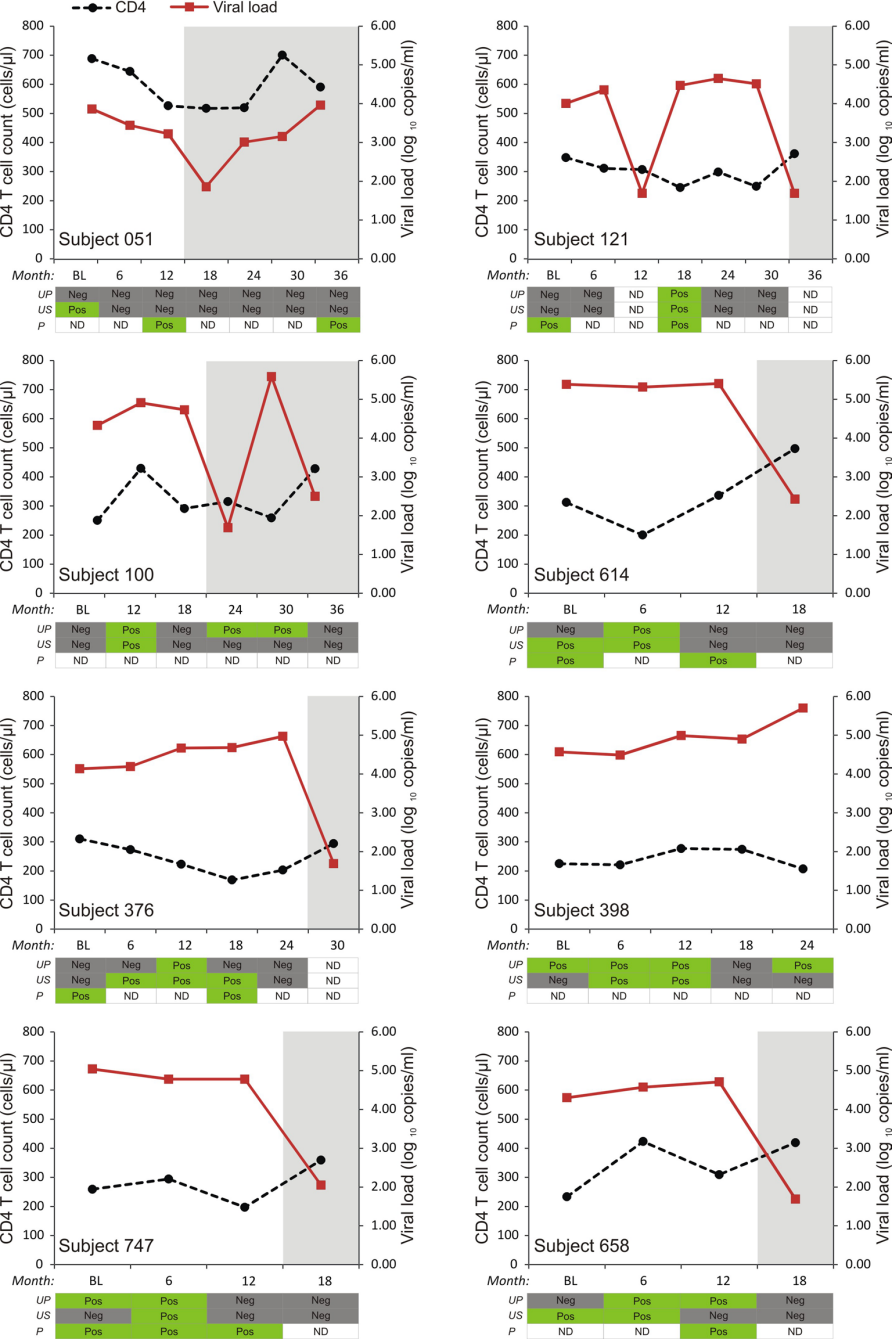
CD4 T cell counts and viral loads over time in subjects showing detection of HIV nucleic acid in urine samples and plasma. Y-axis left: CD4 (cells/μl), black dotted line; Y-axis right: viral load (log_10_ copies/ml), red line. Subject IDs: Nucleic acid was detected in urine supernatant and pellet samples in all the subjects except Subject 051. In Subject 100, nucleic acid was detected in urine pellet during treatment period including period of virological failure (month 30) and period of undetectable viremia in blood (month 24). *BL* baseline (enrolment) sample, *UP* urine pellet, *US* urine supernatant, *P* plasma. Positive (Pos) samples are highlighted in green, negative (neg) in grey, *ND* not determined. Grey-shaded graph regions denote period of antiretroviral therapy

To determine whether the detection of HIV-1 DNA in urine pellets associated with the amount of cells present in the urine, we compared the quantity of isolated genomic DNA (directly proportional to cell number) between urine pellet samples with and without detectable HIV nucleic acid. The mean DNA quantity was higher for urine pellets with detectable HIV nucleic acid compared to those with no detectable HIV nucleic acid (1.81 micrograms versus 0.37 μg; P = 0.01). For all urine samples, the frequency of detectable HIV RNA in supernatants and HIV proviral DNA in pellets were similar (13.4% and 14.4%, respectively). However, the frequency of detection for each individual over time varied substantially (Fig. 1).

### HIV-1 nucleic acid detection in urine samples during treatment period

To investigate if HIV detection in urine samples persists during treatment with antiretroviral therapy, we analysed urine samples obtained after treatment initiation. There were 26 urine samples tested (13 supernatant samples and 13 urine pellets) from 7 subjects that corresponded with periods of virological failure (VL > 1000 RNA copies/ml) in blood (Fig. 1). HIV-1 nucleic acid was detected in 1 urine pellet from subject 100. HIV-1 *pol* gene sequencing of the corresponding plasma samples did not detect resistance-associated markers from these samples. There were 42 samples tested (21 supernatant samples and 21 urine pellets) from 13 subjects that corresponded with periods of either undetectable viremia (Limit of detection 40 RNA copies/ml) or detectable viremia < 1000 RNA copies/ml in blood. HIV nucleic acid was detected in 1 urine pellet from subject 100 obtained more than 6 months after therapy initiation and during a period of undetectable viremia in blood.

### Comparison of HIV-1 viral populations in urine to variants in blood

**Fig. 2:**
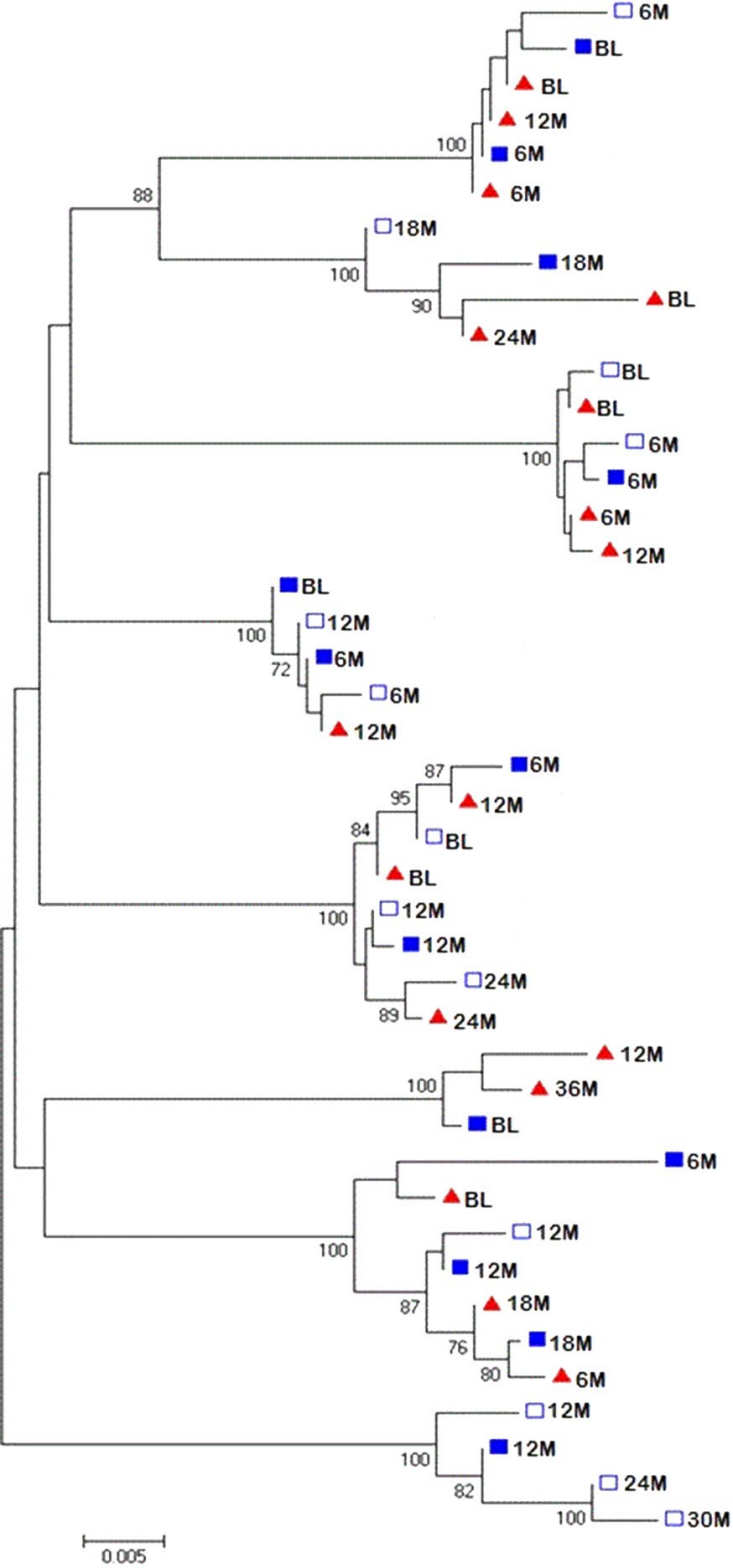
Phylogenetic tree of the sequenced *pol* gene from the urine and blood viruses. The evolutionary history was inferred using the Neighbour-Joining method. The bootstrap values (1000 replicates) are shown next to the branches. The evolutionary distances were computed using the Kimura 2-parameter method. Evolutionary analyses were conducted in MEGA6. The sequence clusters from the 8 subjects with HIV-1 nucleic acid detected in urine are indicated. Red triangles—plasma RNA, blue squares—urine supernatant variants, empty blue squares—urine pellet variants. Sequences are named using time points (e.g. 6 M = 6 months, *BL* baseline)

To compare HIV-1 viral populations in the urine to viruses in blood, a phylogenetic tree was constructed using the *pol* sequences obtained from the urine (supernatant and pellet samples) and from plasma (Fig. 2). Sequences included in the tree were from viruses from individuals where two or more sequences were obtained from urine samples. Co-clustering of urine-derived *pol* sequences among the plasma-derived sequences was observed while some urine-derived sequences were interspersed among the plasma-derived sequences. The co-clustering of urine-derived sequences in these individuals occurred between pellet-derived provirus sequences and sequences from free viruses in the urine supernatant. This is demonstrated in subject 747 for the 6 month sequences (6 M), subject 398 for the 12 month sequences (12 M) and in subject 376 for the 12 month (12 M) sequences (Fig. 2). Interspersing of urine-derived and plasma-derived sequences is shown in most of the sequences exemplified by urine-derived sequence 18 M and plasma-derived 6 M sequence which clustered.

## Discussion

We analysed urine samples for HIV-1 shedding in the urinary tract before and after suppressive ART initiation. We have also investigated HIV-1 replication in the urinary tract, evidenced by detection of HIV-1 RNA in treatment naïve individuals, and HIV-1 compartmentalization by comparing HIV sequences from plasma-derived viruses to sequences derived from viruses in urine samples using phylogenetic analysis. We detected HIV-1 nucleic acid in urine samples from 40% of treatment-naïve subjects compared to 8% in the virally suppressed or low level viremia group and 14% in the virological failure group. Although phylogenetic analysis indicated co-clustering of provirus sequences and sequences from free viruses in the urine supernatant a larger number of sequences will be required to draw conclusions regarding virus compartmentalization in the urinary tract. We also detected viruses related to those circulating in blood resulting in equilibrium between blood and urinary tract viruses.

The low HIV-1 nucleic acid detection rates in urine samples from individuals on suppressive ART compared to treatment-naïve individuals indicates that suppressive ART is effective in blocking viral replication in the urinary tract. HIV-1 nucleic acid detection in urine samples from HIV positive treatment-naïve individuals has been reported in other studies [17, 18], followed by undetectable levels after treatment initiation [18]. Our data adds more information in that our cohort had a different subtype (C compared to B) and we analysed longitudinal samples instead of a single time point. In addition we looked at the *pol* gene instead of the *env* gene.

We detected HIV-1 RNA in urine pellets more than 6 months after treatment was initiated highlighting that infected cells can be shed into the urinary tract during periods of both viral suppression and virological failure in blood. The individual had virological failure after a period of viral suppression in blood with detectable HIV-1 DNA in cells shed in urine. In another study, HIV-1 DNA was detected in the urine samples in 23% of virally suppressed subjects on ART [19]. This raises the question whether HIV-1 DNA in cells of the urinary tract is replication-competent and may be responsible for viral rebound upon treatment cessation or virological failure during treatment. HIV-1 infection of kidney allografts after transplantation in HIV positive recipients with undetectable HIV-1 virus in plasma has been reported [14] indicating that this HIV-1 DNA may be replication competent and the source of infection in renal transplants.

In individuals with detectable HIV-1 nucleic acid in urine, we noted that detection of the HIV-1 did not occur at all the time points tested regardless of the associated higher plasma viral loads. The frequency of detection for each individual over time varied substantially (Fig. 1), which suggests that specific factors may modulate the presence of HIV in urine. Firstly, HIV-1 shedding in the urogenital tract may be intermittent leading to absence of virus in some samples. Secondly, HIV detection may depend on the urine volumes used. Urine volumes of 2 ml were used for nucleic acid extraction in this study. Larger urine volumes resulted in higher HIV-1 RNA [13] and HIV-1 proviral DNA [17] detection rates. HIV-1 RNA detection rate was 50% in samples with a starting volume ranging from 35 to 630 ml [13] while there was HIV-1 DNA detection rate of 66.25% achieved using cell pellets obtained from 100 ml fresh urine samples [17]. Finally, the presence or degree of renal injury, which may affect the rate of shedding of renal epithelial cells, was unknown in our subjects. This information could help determine why HIV-1 nucleic acid could be detected in some individuals or at certain times as HIV-associated nephropathy has been associated with HIV-1 replication and compartmentalization [9, 19]. We however used the amount of DNA in urine pellets after nucleic acid extraction as an indicator of presence and amount of cells shed into the urogenital tract. Our results indicated that urine samples with detectable HIV-1 DNA had significantly higher numbers of cells (larger amount of host DNA extracted) compared to urine samples with no detectable HIV-1 nucleic acid which may be an indication of renal injury and HIV-1 replication at the time of sample collection. Although HIV-1 virus could have been intermittently shed in urine for some individuals, HIV-1 RNA and proviral DNA were consistently not detected in longitudinal urine samples taken over 18 months from some treatment-naïve individuals.

In conclusion, we have demonstrated that urine samples can be used as a non-invasive method to study HIV replication, persistence and compartmentalization in the urinary tract. Suppressive ART reduces HIV-1 replication in the urinary tract but HIV-1 DNA can persist and be shed from some cells in the urinary tract despite treatment. Our data suggests HIV-1 replication in the urinary tract but also direct virus importation from blood resulting in equilibrium between blood viruses and viruses shed from the urinary tract but a larger number of sequences would be required to confirm compartmentalization.

## Supplementary information

**Additional file 1: Table S1.** Study subjects and time points at which urine samples were tested.

## Data Availability

Data generated or analysed during this study are included in this published article and its additional file. Additional datasets used and/or analysed during the current study are available from the corresponding author on reasonable request.

## Abbreviations

DNA: deoxyribose nucleic acid
ART: antiretroviral therapy
RNA: ribonucleic acid
PR: protease
RT: reverse transcriptase
Pol: polymerase
VL: viral load
RT-PCR: reverse transcription polymerase chain reaction
