# Supplementary material for: Detection and molecular characterization of urinary tract HIV-1 populations
Source: Ann Clin Microbiol Antimicrob. 2019 Sep 24;18:27. doi: 10.1186/s12941-019-0326-9 (PMC6760049; doi:10.1186/s12941-019-0326-9)
Supplement: Supplementary file 1 — Additional file 1: Table S1. Study subjects and time points at which urine samples were tested. [file 12941_2019_326_MOESM1_ESM.docx]

**Table S1: - Study subjects and time points at which urine samples were tested**

| **Subject ID** | **Gender** | **Age** | **VL (RNA copies/ml/ CD4 count (Cells/µl) in blood** | | | | | | |
| --- | --- | --- | --- | --- | --- | --- | --- | --- | --- |
|  |  |  | **Month** | | | | | | |
|  |  |  | **0** | **6** | **12** | **18** | **24** | **30** | **36** |
| **030** | Male | 39 | 11696/ 270 | 7936/ 226 | 9405/ 182 | 11549/ 229 | BDL/ 332 | BDL/ 286  (X) | BDL/ 305  (X) |
| **038** | Female | 42 | 13486/ 361 | 9107/ 167 | 21990/ 92 | 1493/ 190 | 10892/ 115 | 49514/ 203 | 31952/ 101 |
| **051^φ^** | Female | 34 | 7304/ 688 | 2757/ 644 | 1672/ 526 | 72/ 517 | 1019/ 519 | 1427/ 700 | 9187/ 590 |
| **074** | Female | 36 | 14392/ 242 | X | 163536/ 173 | X | X | X | BDL/ 444 |
| **100*^φ^** | Female | 28 | 21289/ 250 | X | 81236/ 429 | 53392/ 291 | BDL/315 | 383953/ 259 | 315/ 428 |
| **106** | Female | 42 | 8287/ 186 | 10630/ 200 | 7399/ 107 | X | 5605/ 106 | 74494/ 76 | BDL/ 165  (X) |
| **121** | Male | 40 | 35750/ 136 | BDL/ 253 | 115/ 175 | 45050/ 161 | BDL/ 163 | BDL/ 293 | 7118/ 173 |
| **142^φ^** | Female | 31 | 10145/ 348 | 22565/ 311 | X | 29474/ 245 | 44596/ 298 | 32422/ 249 | BDL/ 361  (X) |
| **203** | Female | 35 | 20643/ 337 | 16606/ 200 | 12830/ 344 | 25961/ 315 | 23948/ 241 | 30106/ 238 | X |
| **236** | Female | 41 | 86005/ 296 | 38888/ 385 | 12063/ 245 | 68335/ 736 | BDL/ 572 | 53/ 574 | X |
| **376^φ^** | Male | 48 | 13570/ 310 | 15532/ 273 | 46493/ 223 | 47901/ 169 | 93889/ 203 | BDL/ 294 | X |
| **398^φ^** | Female | 33 | 37227/ 225 | 30717/ 221 | 97824/ 277 | 79422/ 274 | 500000/ 207 | X | X |
| **614^φ^** | Female | 33 | 242784/ 312 | 206259/ 200 | 254093/ 336 | 265/ 497 | X | X | X |
| **658^φ^** | Female | 37 | 19983/ 233 | 37512/ 423 | 51229/ 309 | BDL/ 419 | X | X | X |
| **662** | Female | 30 | 10655/ 273 | 11248/ 200 | 9874/ 176 | X | X | X | X |
| **664** | Female | 34 | 15736/ 258 | BDL/ 385 | 39078/ 364 | BDL/ 325 | X | X | X |
| **668** | Female | 54 | 13525/ 420 | 80/ 476 | 127/ 548 | BDL/ 535 | X | X | X |
| **705** | Female | 35 | 412812/ 231 | 345938/ 254 | 61/ 376 | BDL/ 332 | X | X | X |
| **732** | Female | 39 | 15405/ 225 | 6294/ 233 | BDL/ 344 | BDL/ 364 | X | X | X |
| **747^φ^** | Female | 45 | 110279/ 259 | 60362/ 294 | 60306/ 197 | 112/ 359 | X | X | X |

VL-viral load, BDL – below detection limit (40 RNA copies/ml)

^φ^Subjects with HIV-1 nucleic acid detected in urine samples, *Plasma samples not available, Grey shading – period subject on antiretroviral therapy, X – Sample not available at indicated period, X in grey shaded area – Sample not available at indicated time point
